# Biocontrol efficacy of *Bacillus velezensis* strain YS-AT-DS1 against the root-knot nematode *Meloidogyne incognita* in tomato plants

**DOI:** 10.3389/fmicb.2022.1035748

**Published:** 2022-11-22

**Authors:** Yanfeng Hu, Jia You, Yu Wang, Yong Long, Siru Wang, Fengjuan Pan, Zhenhua Yu

**Affiliations:** ^1^Key Laboratory of Mollisols Agroecology, Northeast Institute of Geography and Agroecology, Chinese Academy of Sciences, Harbin, China; ^2^Institute of Pratacultural Science, Heilongjiang Academy of Agricultural Science, Harbin, China; ^3^University of Chinese Academy of Sciences, Beijing, China

**Keywords:** *Bacillus velezensis*, biocontrol agent, induced systemic resistance, root-knot nematodes, plant-parasitic nematodes, *Meloidogyne incognita*

## Abstract

Root-knot nematodes (RKNs; *Meloidogyne* spp.), one of the most economically important plant-parasitic nematodes (PPNs), cause severe yield and quality losses in agriculture annually. The application of biological control agents is an environmentally safe and effective approach to control RKNs. Here, we report the genomic characteristics of a *Bacillus velezensis* strain YS-AT-DS1 (*Bv-DS1*) isolated from the tidal soil, revealing that it has a 4.73 Mb circular chromosome with an average GC-content of 46.43%, 3,977 genes, 86 tRNAs, and 27 rRNAs, and contains secondary metabolite clusters for producing antimicrobial compounds. *In vitro* assays indicated that *Bv-DS1* has not only antagonistic activities against fungal pathogens, but also shows nematicidal activity, with a mortality rate of 71.62% mortality rates in second-stage juvenile (J2s) *Meloidogyne incognita*. We then focused on the biocontrol efficiency of *Bv-DS1* against *M. incognita* in pot assays. Preinoculation with *Bv-DS1* enhanced tomato growth, and significantly reduced the infection rate of J2s, and the number of galls and egg masses on tomato roots. The underlying mechanism in *Bv-DS1-*induced resistance to *M. incognita* was further investigated through split-root experiments, and analysing the expression of the genes related to jasmonic acid (JA), salicylic acid (SA), and the tonoplast intrinsic protein (TIP). The results indicated that *Bv-DS1* could not activate host systemic-induced resistance (ISR) in the split-root system of tomatoes. Additionally, the expression of JA- (*LOX D* and *MC*) and SA- (*PAL2* and *PR*) responsive genes did not change in *Bv-DS1*-pretreated plants at 3 and 14 days after nematode inoculation. The presented data showed that JA-and SA-dependent pathways were not required for the biocontrol action of the *Bv-DS1* against RKN. The *TIP* genes, responsible for transport of water and small substrates in plants, have previously been shown to negatively regulate the parasitism of PPNs. Surprisingly, *Bv-DS1* compromised the downregulation of *TIP1.1* and *TIP1.3* by *M. incognita*. Together, our data suggest that *Bv-DS1* exhibits a dual effect on plant growth promotion and protection against RKN, possibly related to the regulation of water and solute transport *via TIPs*. Thus, the *Bv-DS1* strain could be used as a biocontrol agent for RKN control in sustainable agriculture.

## Introduction

Root-knot nematodes (RKNs; *Meloidogyne* spp.) are the most economically important plant-parasitic nematodes (PPNs) that cause severe yield losses of at least 100 billion dollars annually (Elling, 2013). As obligate biotrophs, RKNs have a broad range of host plants and are parasitic to more than 5,000 plant species, including field crops, vegetables, grass shrubs, and even fruit trees (Blok et al., 2008). RKNs have a very short life cycle, high reproductive capacity, and mainly attack the roots of growing plants. In addition, RKN infection, in combination with other fungal and bacterial pathogens in the soil, can cause secondary damage to host roots, which further exacerbates crop loss (Jones et al., 2013). In China, RKNs have also become a major yield-limiting factor in protected agriculture due to intensive production, continuous monoculture, and the maintenance of a stable microclimate (Jin et al., 2017, 2022). For example, RKNs can often be found during the off-season in polyhouse cultivation of vegetables in the northeast and northwest China due to favourable conditions such as moisture, temperature, and continuous availability of hosts, causing severe economic losses (Li K. et al., 2015, 2017; Liang, 2017; Li, 2020). Common management methods for RKNs include the utilisation of synthetic chemical nematicides and RKN-resistant cultivars (Giannakou and Anastasiadis, 2005; Jordan, 2018). However, breeding resistant cultivars requires lengthy procedures and heavy manpower, and there are limited genetic resources to develop resistance to RKNs (Davies and Elling, 2015). Frequent and excessive application of the synthetic chemical nematicides has caused high toxicity to soil ecosystems and humans, resulting in severe restriction or outright bans (Aktar et al., 2009; Coyne et al., 2018). Thus, there is an urgent need to explore environmentally safe and effective alternatives to control RKNs.

Biological control has emerged as an environmentally-friendly alternative to suppress various soil-borne pathogens, including PPNs (Mhatre et al., 2019; Lahlali et al., 2022). Numerous microorganisms, including fungi, bacteria, and actinomycetes, have been identified as potential biocontrol agents for the efficient management of RKNs in many crops, especially vegetables (Silva et al., 2017; Kim et al., 2018; Jin et al., 2019; Park et al., 2020; Pocurull et al., 2020; Sharma et al., 2020; Shahid et al., 2022; Wang et al., 2022). Some microbial antagonists of RKNs are able to directly parasitize nematode eggs or other developmental stages, such as *Streptomyces rubrogriseus*, *Pasteuria penetrans* (Davies and Curtis, 2011; Jin et al., 2017; Topalović et al., 2019). Some fungal and bacterial species were reported to produce metabolites which indirectly reduce RKN density by inhibiting egg hatching, repelling, immobilizing and/or killing J2s (Cheng et al., 2017; Park et al., 2020; Khoja et al., 2021; Sun et al., 2021). In addition, induction of resistance in plants by these biocontrol microorganisms is another indirect strategy for controlling RKNs (Dehghanian et al., 2020; Pocurull et al., 2020; Sharma et al., 2020). Other microbial species have often shown versatility in the mechanisms of control of RKNs. For instance, several species of *Pasteuria* spp. and *Pochonia* spp. exhibited parasitism against eggs and sedentary stages of RKNs, and can also produce secondary metabolites with nematicidal activity or activate plant defences against RKNs (Selim et al., 2014; Giné et al., 2016; Ghahremani et al., 2019).

Rhizosphere bacteria belonging to the *Bacillus* genus have been widely described to effectively reduce RKNs in both greenhouse and field experiments, such as *B. firmus* (Terefe et al., 2009), *B. pumilus* (Lee and Kim, 2016), *B. amyloliquefaciens* (Jamal et al., 2017), *B. subtilis* (Cao et al., 2019; Das et al., 2021), *B. atrophaeus* (Ayaz et al., 2021), *B. cereus* (Yin et al., 2021b) and *B. altitudinis* (Ye et al., 2022). As *Bacillus* species can rapidly colonise and reproduce in the plant rhizosphere and exhibit strong resistance to various environmental stresses, biocontrol agents based on *Bacillus* have shown greater advantages in production, storage, and reliability of RKN biocontrol efficiency compared with other bacterial antagonists of RKN (Lalloo et al., 2010). Moreover, *Bacillus* species can enhance plant growth and improve plant health. Some *Bacillus* strains have been commercially approved in many countries to control PPNs in agriculture, such as *B*. *subtilis* GB03 (Kloepper et al., 2004), *B. firmus* GB-126 (Wilson and Jackson, 2013) and *B. firmus* I-1582 (European Commission, 2019). The *Bacillus* genus achieve their biocontrol effectiveness against RKN through different mechanisms, including parasitism, production of nematicidal chemicals, intoxication, induction of plant systemic resistance, and regulation of water and nutrient uptake. For example, *B. firmus* I-1582 was proved to colonize eggs of *M. incognita* and degrade eggshells (Ghahremani et al., 2020). Microbial community analysis of infected J2s of RKNs showed a dominance of the *Bacillus* genera in suppressive soil against RKNs (Adam et al., 2014), suggesting some *Bacillus* species might parasitize J2s of RKN. *B. thuringiensis* has been found to produce Cry proteins that result in lysis of the intestine and nematode death (Wei et al., 2003). Other *Bacillus* isolates are reported to produce volatile organic compounds to prevent plant roots from RKN invasion by increasing mortality, reducing motility, or inhibiting hatching of J2s from the eggs (Huang et al., 2010; Gao et al., 2016; Du et al., 2017; Chen et al., 2021; Yin et al., 2021a; Ye et al., 2022). Previous studies have also provided evidence that the induced systemic resistance (ISR) in host plants by *Bacillus* species contributes to their antagonistic effects against RKNs (Ayaz et al., 2021; Yin et al., 2021b; Tian et al., 2022). It is worth noting that the activation of specific plant signalling pathways during ISR by *Bacillus* spp. varies depending on the species of bacterial isolates, host plant, and nematode. Additionally, El-Hadad et al. (2011) demonstrated that the inoculation of *B. megaterium* can suppress the population of RKN in the soil through regulation of phosphate solubilisation and mineralisation capacity. *B. cereus BCM2* was verified to have excellent nematicidal activity against RKNs by secreting proteases (Hu et al., 2020). Thus, the identification of more antagonists from *Bacillus*. spp. is essential for their mass production and application in integrated strategies for RKNs control. While a comprehensive understanding of nematode biocontrol mechanisms using the genera *Bacillus* is a pre-requisite for further improving biocontrol efficiency of RKNs in agriculture.

Here, the *B. velezensis* strain YS-AT-DS1 (*Bv-DS1*) strain was previously isolated from tidal soil in Dongying city (Shandong province, China), and displayed promoted growth and antagonistic effects on pathogenic fungi. Thus we speculated it might have nematicidal activities against *M. incognita*. The main objective of this study was to investigate the biocontrol activity of *Bv-DS1* against *M. incognita* in pot assays. The capability of *Bv-DS1* to induce systemic resistance against *M. incognita* was determined in split-root system of tomato. To better understand the potential biocontrol mechanism, the expression of defence-related genes involved in jasmonic acid (JA), salicylic acid (SA), and ion-transport pathways were analysed in *Bv-DS1-*inoculated roots of tomato after nematode infection. In addition, tomato SA and JA mutant lines were used to assess the importance of the hormone-mediated defence pathways in biocontrol effects.

## Materials and methods

### Plant materials and nematode culture

Wild-type tomato (*Solanum lycopersicum*) cultivars “Castlemart” and “Moneymaker”, the JA biosynthetic mutant *spr2* in the “Castlemart” cultivar (Li et al., 2003), and the SA-deficient *NahG* transgenic line in the “Moneymaker” cultivar were kindly provided by Prof. Zhao Jiuhai (Northeast Institute of Geography and Agroecology, Chinese Academy of Sciences). The susceptible tomato cultivar “Zhongshu-4” was used in all nematode inoculation assays, unless indicated otherwise. Tomato seeds were surface sterilised in 1% NaClO for 5 min and then rinsed thoroughly with sterile water three times. The seeds were germinated in sterile vermiculite for 5–7 days at 26°C and then maintained in a growth chamber with a photoperiod of 16-h light (26°C) and 8-h dark (21°C). After 3 weeks of growth, tomato plants were used for nematode inoculation. All plants were watered daily and fertilised twice per week with Hoagland solution.

The population of *Meloidogyne incognita* used in this study was cultivated on the tomato cultivar “Zhongshu-4” (susceptible to *M. incognita*) in a greenhouse with a 16/8-h light/dark cycle at 21–26°C. Egg masses were extracted from tomato roots on the 42nd day after inoculation. Eggs were collected on a 25 μm sieve and placed in an incubator at 28°C for hatching second-stage juveniles (J2s). Fresh J2s were collected daily and used as inoculums for testing nematode mortality and infection assays.

### Strain isolation, identification, and genomic of DNA extraction

*Bv-DS1* was isolated from a tidal soil sample collected in Dongying, Shandong Province, China, using the 10-fold dilution method on lysogeny broth (LB) medium. The complete 16S rRNA was sequenced in BGI (Shenzhen, China) and blasted using EzBioCloud (https://www.ezbiocloud.net/). The purified strain was stored in the China centre for type culture collection (CCTCC) with the accession number CCTCC M 2021239. Genomic DNA was extracted using the blood and cell culture DNA midi Kit (Cat. No. 13343, Qiagen, United States) according to the manufacturer’s protocol. Briefly, an appropriate volume of cultured bacteria was pelleted by centrifugation at 4,000 *× g* for 10 min and the supernatant was discarded. The bacteria pellet was then resuspended in 3.5 ml of Buffer B1 (with RNase A) by vortexing at top speed. A stock solution of 20 μl lysozyme stock solution (100 mg/ml) and 100 μl QIAGEN Protease or QIAGEN Proteinase K was added and incubated at 37°C for at least 30 min. Next, 1.2 ml of Buffer B2 was added and mixed by vortexing for a few seconds, followed by incubation at 50°C for 30 min. Then, the sample was vortexed for 10 s at maximum speed and applied to the equilibrated QIAGEN Genomic-tip. The QIAGEN Genomic-tip was washed with 2 × 7.5 ml of Buffer QC, followed by the precipitation, purification and dissolving of DNA. DNA concentration and purity were determined using a Qubit fluorometer and NanoDrop 2000 spectrophotometer (Thermo Fisher Scientific, Carlsbad, CA, United States). DNA integrity was assessed using 0.5% agarose gel electrophoresis.

### Library construction and sequencing of the *Bv-DS1* genome

Whole genome sequencing was performed using the MGISEQ-2000 platform and Oxford Nanopore Technologies (ONT) PromethION P24 device at BGI (Shenzhen, China). For the MGI sequencing library, the insert size was 350 bp with a pair-end sequencing length of 150 bp. Briefly, 1 μg of genomic DNA was randomly fragmented using a g-TUBE device (Covaris, Inc., Woburn, MA, United States) according to the manufacturer’s instructions. The DNA fragments with an average size of 200–400 bp were selected using magnetic beads. The selected fragments were 3′-adenylated through end-repair and adapter-ligation; PCR products were purified using the magnetic beads. The double-stranded PCR products were heat denatured and circularised using the splint oligo sequence. The single-strand circular DNA (ssCirDNA) was formatted as the final library and qualified using FastQC. For ONT sequencing, genomic DNA was used to construct a library using a ligation sequencing kit (SQK-LSK109) and native barcoding kit (EXP-NBD114) according to the standard 1D native barcoding protocol provided by the manufacturer (Oxford Nanopore, Oxford, UK). Briefly, 48 μl of genomic DNA was mixed with 3.5 μl NEBNext FFPE DNA repair buffer (New England BioLabs, Ipswich, MA, United States), 2 μl NEBNext FFPE DNA repair mix (NEB), 3 μl ultra II end-prep enzyme mix (NEB), and 3.5 μl ultra II end-prep reaction buffer (NEB) in a 200 μl PCR tube. The mixture was incubated at 20°C for 5 min followed by 65°C for 5 min. Next, 500 ng end-prepped samples were mixed with 2.5 μl native barcode (one barcode per sample) and 25 μl blunt/TA ligase master mix. The mixtures were incubated at 28°C for 10 min. A total of 700 ng pooled and barcoded DNA was used to perform adapter-ligation by adding 20 μl NEB next quick ligation reaction buffer (5×), 5 μl adapter mix II and 10 μl quick T4 DNA ligase. The mixture was incubated for 10 min at room temperature. The constructed library was quantified using a Qubit DNA HS assay kit in a 4.0 Fluorometer (Invitrogen, San Diego, CA, United States) and then loaded into the flow cell R9.4.1 of a PromethION P24 device (BGI-ShenZhen, China).

### Genomic data analysis of *Bv-DS1*

All the raw data were trimmed using SOAPnuke v.1.5.2 (Li D. et al., 2015). High-quality reads were assembled *de novo* using Megahit software (Chen et al., 2018). Assembled contigs with lengths less than 300 bp were discarded in the subsequent analysis. The prediction of coding genes (CDS) was analysed with Glimmer (version 3.02) and the annotation was done by alignment against the COG, Gene Ontology (GO), and Kyoto Encyclopedia of Genes and Genomes (KEGG) databases. Secondary metabolites analysis was performed using antiSMASH 5.0 software (Blin et al., 2019).

### Inhibition of pathogenic fungi

The ability of *Bv-DS1* to inhibit *Rhizoctonia solani*, *Fusarium avenaceum* and *Fusarium graminearum* were investigated using a plate confrontation method according to the description by Gao et al. (2021). Briefly, the pathogenic fungi were inoculated in the centre of the PDA plate and 100 μl *Bv-DS1* (OD_600_ = 1) were inoculated 2.5 cm from the centre containing pathogenic fungi, with plates not inoculated with *Bv-DS1* used as a control. All plates were incubated at 27°C for several days until the pathogenic fungi on the control plates grew all over the petri dish. Then the growth diameter of the pathogen was measured.

### Screening the ability of IAA production

The measurement of IAA production was conducted by using a modified quantification method based on Bric et al. (1991). Briefly, *Bv-DS1* was cultured for 24 h in 1 ml of LB liquid medium. Then 10 μl of bacterial inoculums were transferred into the same medium supplemented with 100 μg mL^−1^ of L-tryptophan (Sigma-Aldrich) followed by 7 days of incubation at 28°C on a shaking incubator (200 rpm/min). Then, bacterial cells were removed from the culture medium by centrifugation (4,000 *× g*, 5 min). The supernatant was then transferred with Salkowski reagent (49 ml of 35% HClO_4,_ 1 ml of 0.5 M FeCl_3_) to an ELISA plate in a 1:1 the ratio, which was incubated at room temperature for 35 min. The absorbency was then read at 490 nm by using a multi-functional enzyme labeller (CLARIOstar Plus, BMG, Germany). The uninoculated tryptophan-containing medium mixed with Salkowski reagent was used as a blank. Three independent cultivations were used as triplicated replicates. A standard curve was generated from serial dilutions of IAA stock solution.

### Effect of *Bv-DS1* culture filtrate on J2s mortality of *Meloidogyne incognita*

*Bv-DS1* was cultured in 100 ml of LB liquid medium for 48 h at 28°C on a shaking incubator (200 rpm/min). The fermented bacteria were centrifuged at 2,500 × *g* for 10 min, and the bacterial pellet was resuspended and adjusted to a density of 1.0 × 10^8^ colony-forming units (CFUs) per millilitre with sterile water, which was used further for inoculation. Additionally, the supernatant of the *Bv-DS1* strain was collected and filtered using a 0.22-μM Millipore filter. The prepared culture filtrate was used to test nematocidal efficacy *in vitro*.

For inoculation with *Bv-DS1*, the 3-week-old tomato plants were transplanted to individual pots (14 cm in height and 12 cm in diameter) filled with sterilized sand-soil medium (2:1, vol/vol) for another 3 days until the initiation of the experiment. Each transplanted plant was inoculated with *Bv-DS1* by adding 20 ml of bacterial suspension into 2-cm-deep holes. Subsequently, the plants were put back in the growth chamber and used for nematode inoculation after 3 days of growth.

For nematode mortality assay, 10 μl of nematode suspension (approximately 100 J2s) and 490 μl of Bv*-DS1* culture filtrate (100 and 10%, respectively) were added to each well of the 24-well culture plate (Corning, United States), and sterile water was used as the control. The plates were incubated in darkness for 24 h at room temperature, and then the number of living and dead nematodes were counted under a stereomicroscope (Olympus, Japan). J2s were considered to be dead if their body was straight and immobile after the Na_2_CO_3_ stimulus for 30 s (Hu et al., 2019). The experiment was carried out twice with 10 replicates. Corrected J2s mortality was calculated using the following equation:

[(mortality rate of J2s treated with *Bv-DS1* - mortality rate of J2s treated using the sterile water)/(1 - mortality rate of J2s treated using sterile water)] × 100.

### Nematode inoculation

To investigate the effects of *Bv-DS1* on the nematode-invasion ability, gall formation, and host defence, a pot experiment was conducted at different time points using four treatments: (1) roots treated with sterile water, (2) roots pre-inoculated with *Bv-DS1* alone, (3) roots inoculated with nematodes alone, (4) the roots preinoculated with *Bv-DS1* and then infected with nematodes. Three-week-old tomato plants were transplanted to pots filled with sand-soil medium and were grown for 6 days. Two holes were opened on the surface of pots, and plants were inoculated with 500 J2s per plant in one pot. At 3, 7, and 14 days after nematode inoculation (dai), roots were collected and used for RNA extraction and gene expression analysis. At 35 dai, plant height, fresh weight of root and shoot, and stem thickness were measured, and the disease severity was assessed by counting gall numbers on roots. Each treatment was performed with six replicates, and three independent experiments were conducted for each treatment.

To further evaluate the control efficacy of *Bv-DS1* against RKN in the greenhouse condition, the soil collected from the *M. incognita*-infested tomato field (Shenyang, Liaoning province, China) was used in the pot experiment. The population densities of *M. incognita* were 1.5 nematodes per cm^3^ of soil. Three-week-old tomato plants were transplanted into pots (20.5 cm deep × 14 cm diameter) filled with soil media containing 20% sterilized sand and 80% diseased soil. After 3 days of transplantation, 20 ml of the *Bv-DS1* suspension (10^8^ CFU/ml) was drenched into the rhizosphere of the plant in each pot; the plants were irrigated with the same volume of sterile water that served as the control. All plants were grown in the greenhouse under a completely randomized design for 42 days. Afterwards, the roots of tomato plants were collected, and fresh root weight was measured. Root galls per plant were counted and the number of egg masses was determined using 0.01% erioglaucine (Sigma, St. Louis, MO, United States) staining (Omwega et al., 1988). Finally, eggs were extracted from the roots according to the previously described method (Hussey and Barker, 1973). Each treatment had eight replicates, and the experiment was repeated twice.

### Split-root assay

**Figure 1:**
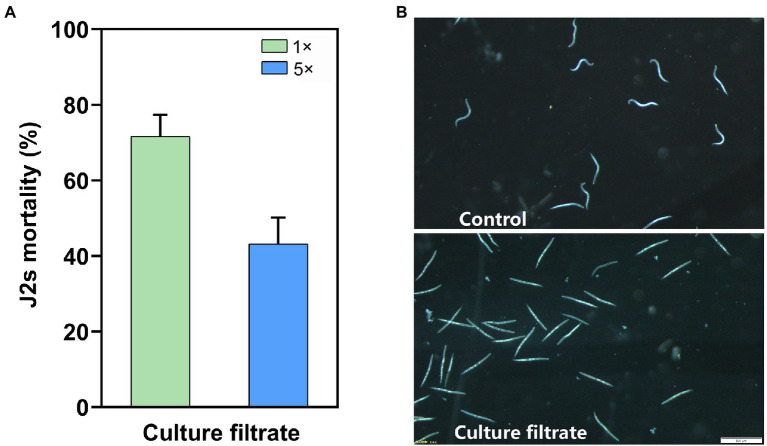
Filtrate culture of *Bacillus velezensis* YS-AT-DS1 (*Bv-DS1*) affected the J2s mortality of *M. incognita*. **(A)** Corrected J2s mortalities of *M. incognita* were analysed after incubation with the 1× and 5× *Bv-DS1* filtrate cultures for 24 h at room temperature. The error bars represent the mean ± SE of the data from 10 replicates. **(B)** Microscopic observation of J2s body immersed in *Bv-DS1* filtrate culture or the sterilized distilled water after 48 h incubation. Scale bar = 500 μm.

The split-root system of tomato was used to evaluate the ability of *Bv-DS1* to induce systemic plant resistance against *M. incognita* as described by Martínez-Medina et al. (2017). Three-week-old tomato plants were transferred to the split-root system by splitting the root system into two halves that were planted into two adjacent pots (14 cm in height and 12 cm in diameter) containing a sterilized sand-soil mixture (2:1, v/v) (Figure 1A). A total of three treatments were used in this experiment with eight replicates for each treatment. The treatments included (1) half of the root system being inoculated with 500 J2s of *M. incognita* (RKN/−) and another half of the roots with sterile water, (2) half of the roots were pre-inoculated with the *Bv-DS1* suspension and then infected with nematodes (RKN + *Bv-DS1*/−), and (3) half of the root system was pre-inoculated with *Bv-DS1* and another half was infected with nematodes (RKN/*Bv-DS1*). For treatments of *Bv-DS1*, the plants in the split-root set-up were inoculated with 20 ml of suspension of *Bv-DS1* (10^8^ CFU/ml) 6 days after transplantation. The pots were placed in the greenhouse under the same condition for another 3 days.

### RNA extraction and quantitative real time-PCR analysis

Tomato roots were frozen in liquid nitrogen and ground to a fine powder in a pestle and mortar. RNA was extracted using an RNAprep pure plant kit (TianGen Biotech, Beijing, China) according to the manufacturer’s instructions. One microgram of total RNA was used to synthesize cDNA using FastKing gDNA dispelling RT SuperMix FastKing Kit (TianGen Biotech, Beijing, China). qRT-PCR analysis was performed in the LightCycler® 480 System with SYBR green master mix (Vazyme, Nanjing, China). The target gene primers used for qRT-PCR are listed in Supplementary Table S1. Reaction conditions were as follows: 95°C for 5 min, and then 40 two-step cycles of 10 s at 95°C and 30 s at 60°C. The relative expression levels of the defence-related tomato genes and the actin gene from *M. incognita* were normalized and calculated using the reference gene expression of *SIEF1α* using the 2^-∆∆Ct^ method.

### Statistical analysis

Data analysis was performed using SPSS version 17.0 software (SPSS Inc., Chicago, United States). The statistically significant differences were analysed using one-way ANOVA (multiple comparisons) or Student’s *t*-test (unpaired comparisons), as shown in the figure legends. The error bars in the figures indicated the standard error (SE) of means, and the significance level was set at *p* < 0.05.

## Results

### Taxonomic identification and genomic features of *Bv-DS1*

**Figure 2:**
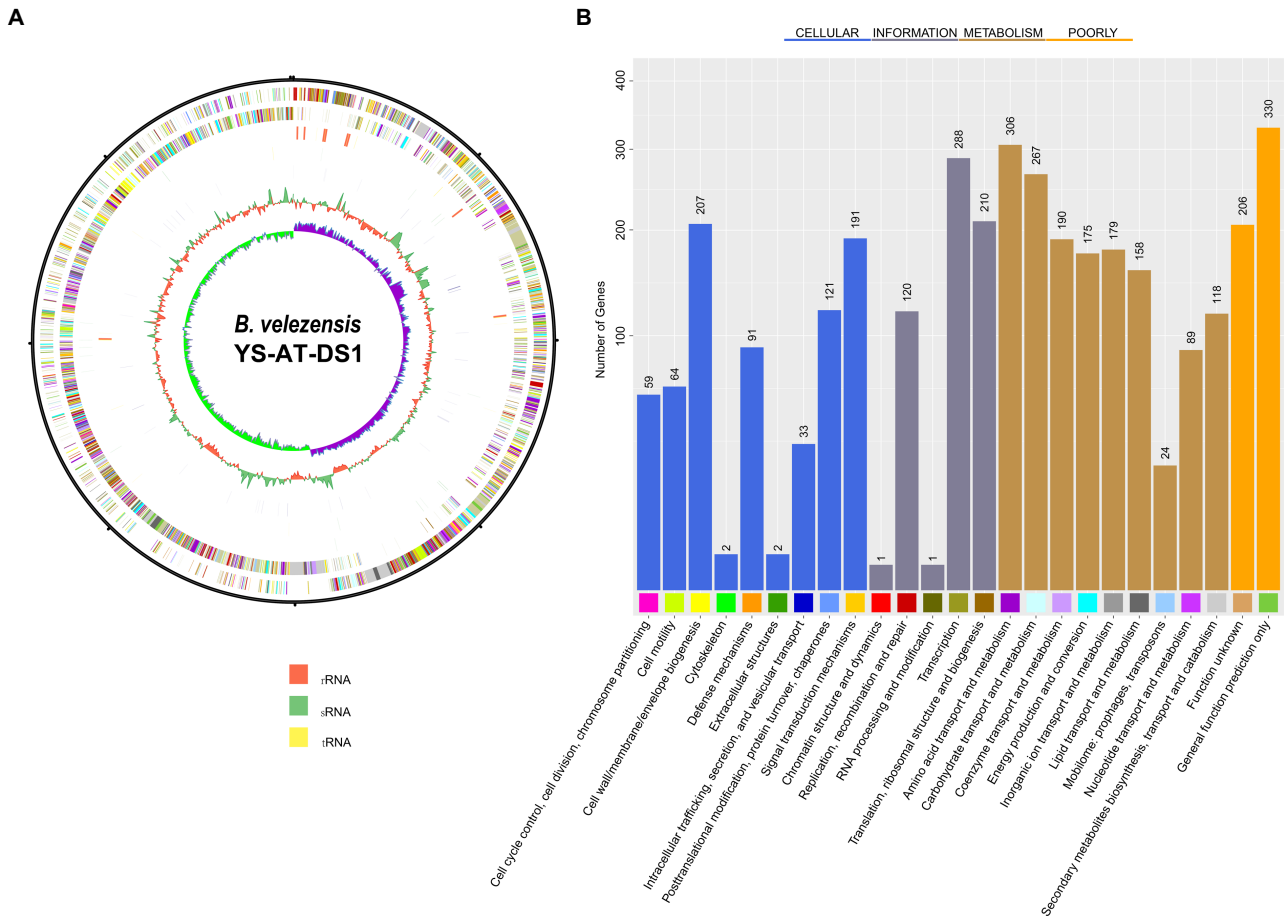
**(A)** Graphical circular map of *Bacillus velezensis* YS-AT-DS1 chromosome. From outer circle to the centre: CDS on forward strand (coloured according to COG categories), all CDS and RNA genes on forward strand, all CDS and RNA genes on reverse strand, CDS on reverse strand (coloured according to COG categories). The map was generated using Bacterial Annotation System, BASys. **(B)** COG functional classification.

Analysing the complete 16S rRNA sequence (1,471 bp) of the stain using EzBioCloud revealed that it showed 100% similarity with *B. velezensis* CR-502. Phylogenetic analysis indicated that it grouped with the *B. velezensis* strain CR-502 T (AY603658), thus confirming its classification as *B. velezensis* (Supplementary Figure 1). Further analysis of genome characteristics revealed that the genome of *Bv-DS1* comprised a circular chromosome of 4.73 Mb (Figure 2A), and was deposited in NCBI with the accession number CP102866. The chromosome of *Bv-DS1* included 4,007,438 bp, with an average GC-content of 46.43%, 3,977 protein-coding genes (CDS), 86 tRNAs, and 27 rRNAs. A total of 4,334 (82.2%) CDSs were classified into Cluster of Orthologous Groups of proteins (COG) families composed of 25 categories (Figure 2B). Among the categories, amino acid transport and metabolism (306 genes), transcription (288 genes), and cell wall/membrane/envelope biogenesis (207 genes) were the top three functional categories. However, there was a high proportion of function unknown genes (206), and general function prediction-only genes (330 genes) were poorly characterized (Supplementary Table S2).

### Genetic basis for the anti-pathogen activity of *Bv-DS1*

Using the antiSMASH genome analysis tool, the detection of secondary metabolite clusters of *Bv-DS1* were detected (Supplementary Table S3). Three trans ATPKS, two NRPS, and one other (bacilysin) cluster showed 100% similarity to the known biosynthetic gene clusters. Several clusters related to surfactin, aurantinin B/aurantinin C/aurantinin D, and butirosin A/butirosin B saccharides were also detected in the *Bv-DS1* genome (Supplementary Table S3).

### IAA production, *in vitro* biocontrol activity of strain *Bv-DS1* against fungal pathogens and root-knot nematodes

As *Bv-DS1* promoted the growth of tomatoes plants, we hypothesized that it might be also able to produce IAA. *Bv-DS1* produced 3.07 μg mL^−1^ IAA and showed antifungal activity against three soybean pathogens (Rhi*zoctonia solani*, *Fusarium avenaceum,* and *Fusarium graminearum*) that cause root rot disease, with the inhibition zones of 1.04 ± 0.18 cm, 1.04 ± 0.15 cm and 1.70 ± 0.20 cm, respectively (Supplementary Figure 2). The nematicidal activity of *Bv-DS1* was assessed in a 24-cell plate by analysing the mortality rates of *M. incognita* J2s after treatment with *Bv-DS1* culture. After incubation for 24 h, the 1 × *Bv-DS1* and 5 × filtrates resulted in 71.62 and 43.16% corrected J2s mortality rates of *M. incognita*, respectively (Figure 1A). The microscopic observation indicated that most J2s were straight and immobile in the filtrate culture of *Bv-DS1* after 48 h treatment. In comparison, the untreated J2s displayed the normal ‘S’ bend shape and were much more active (Figure 1B). These results demonstrated that *Bv-DS1* metabolites had nematicidal activity against *M. incognita in vitro.*

### Promotion of tomato growth and suppression of *Meloidogyne incognita* infection induced by *Bv-DS1*

**Figure 3:**
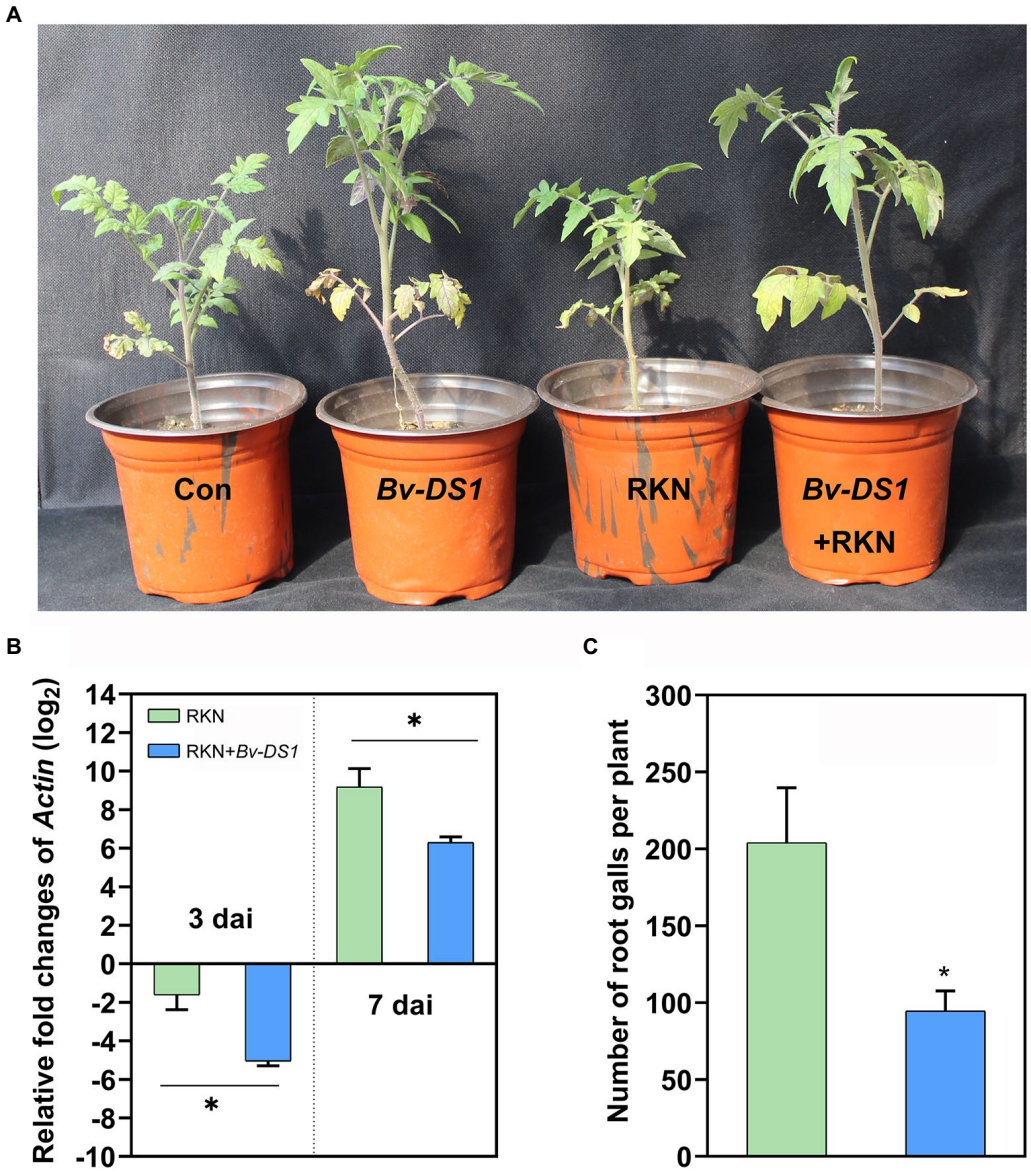
The control efficacy of *Bacillus velezensis* YS-AT-DS1 (*Bv-DS1*) against *M. incognita* in pots. **(A)** Plant growth promotion was observed in the *Bv-DS1*-pre-inoculated tomato plants on the 35th day after inoculation (dai) with *M. incognita*. **(B)** The relative expression folds of the actin gene from *M. incognita* were evaluated inside the roots of *Bv-DS1*-pre-inoculated or non-inoculated tomato plants at 3 and 7 dai. Each value is presented as mean ± SE of three biological replicates for all three plant roots. Asterisks indicate significant differences between treatments (*p* < 0.05) according to Student’s *t* test. **(C)** The number of galls was counted in roots of *Bv-DS1*-pre-inoculated or non-inoculated tomato plants at 35 dai. Data are presented as mean ± SE of eight plants for each treatment. Asterisk indicates statistically significant differences between treatments (*p* < 0.05) according to Student’s *t* test.

The efficacy of *Bv-DS1* against *M. incognita* in tomatoes was evaluated in the pot assay. After 5 weeks of transplantation, *Bv*-*DS1* treatments caused a significant increase in the plant height and root and shoot weight of tomato plants, inoculated or non-inoculated with nematodes, suggesting that *Bv-DS1* had a positive effect on plant growth (Figure 3A; Table 1). The expression of the *actin* gene of *M. incognita* was determined in tomato roots at 3 and 7 dai to investigate the effect of *Bv-DS1* on *M. incognita* infection (Martínez-Medina et al., 2017). Although the *actin* gene of *M. incognita* showed low expression levels in both *Bv-DS1*-treated and untreated plants at 3 dai, *actin* gene expression in *Bv-DS1*-treated roots was significantly lower than that in the non-inoculation roots (Figure 3B). At 7 dai, pre-inoculation with *Bv-DS1* led to a 1.5-fold reduction in expression levels of *actin* compared to the roots without *Bv-DS1* treatments (Figure 3B). These results showed that the application of *Bv-DS1* inhibited early infection of *M. incognita*. Additionally, the number of root galls on tomato roots pre-inoculated with *Bv-DS1* (100 ± 11 per plant) was significantly lower than that of the non-inoculated control roots (200 ± 11 per plant). These results indicated that *Bv-DS1* had plant growth promoting potential and could efficiently control *M. incognita* (Figure 3C).

**Table 1:** Effect of *Bacillus velezensis* YS-AT-DS1 on plant growth parameters after inoculation with RKN.

Treatment	Height (cm)	Stem diameter (cm)	Root weight (FW/g)	Shoot weight (FW/g)
Control	14.68 ± 0.52 b	0.55 ± 0.07a	1.38 ± 0.21 b	2.63 ± 0.20 b
*Bv-DS1*	18.01 ± 0.54a	0.58 ± 0.05 a	2.07 ± 0.11 a	4.19 ± 0.26 a
RKN	14.42 ± 0.66b	0.54 ± 0.06 a	1.30 ± 0.07 b	2.56 ± 0.19 b
*Bv-DS1* + RKN	17.68 ± 0.82 a	0.55 ± 0.04 a	1.89 ± 0.13 a	4.25 ± 0.22 a

Data are presented as mean ± SE of three independent biological replicates of a total of 18 tomato plants for each treatment. Different letters indicate significant differences (*p* < 0.05) according to one-way ANOVA with Tukey’s multiple range test.

### Efficiency of strain *Bv-DS1* against *Meloidogyne incognita* in the disease soil

**Figure 4:**
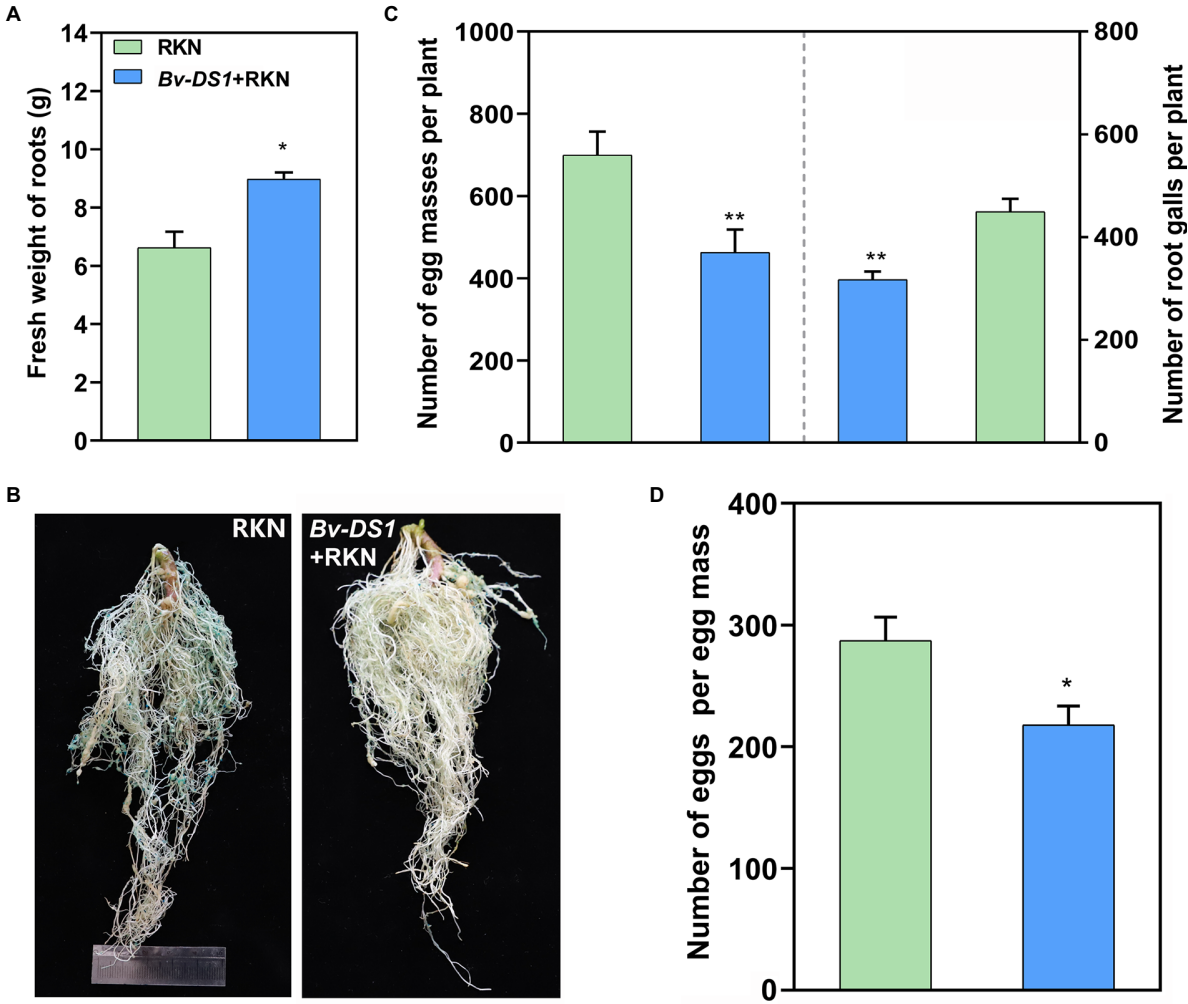
*Bacillus velezensis* YS-AT-DS1 (*Bv-DS1*) reduced *M. incognita* infection when tomato plants were grown in diseased soil. **(A)** Fresh weight of tomato roots pre-inoculated or non-inoculated with *Bv-DS1* growing in the RKN-diseased soil for 42 days. **(B)** Root galling and egg masses on tomato plant pre-inoculated or non-inoculated with *Bv-DS1*. **(C)** Number of galls or egg masses per tomato plant. Data are presented as mean ± SE of 10 plants for each treatment. Asterisk indicates significant differences between treatments (*p* < 0.05) according to Student’s *t* test. **(D)** Number of eggs per egg mass. Data are shown as mean ± SE of eight plants for each treatment. Asterisk indicates significant differences between treatments (*p* < 0.05) according to Student’s *t* test.

The control efficacy of *Bv-DS1* against RKN was tested further in the soil collected from the *M. incognita*-infested tomato field. Irrigation of *Bv-DS1* into the rhizosphere significantly increased the fresh weight of the tomato plants by 35.5% compared to the control group at 42 days (Figure 4A). The number of galls and egg masses were lower on the tomato roots after treatment with *Bv-DS1* (Figure 4B), which decreased by 29.3 and 33.8%, respectively (Figure 4C). Eggs per egg mass from the root system in the soil of *Bv-DS1* drenching was also markedly lower than those collected from the non-inoculated plants (Figure 4D). These results suggested that *Bv-DS1* could enhance the resistance of tomatoes to suppress *M. incognita* reproduction.

### *Bv-DS1* induced the local resistance of tomato against *Meloidogyne incognita*

**Figure 5:**
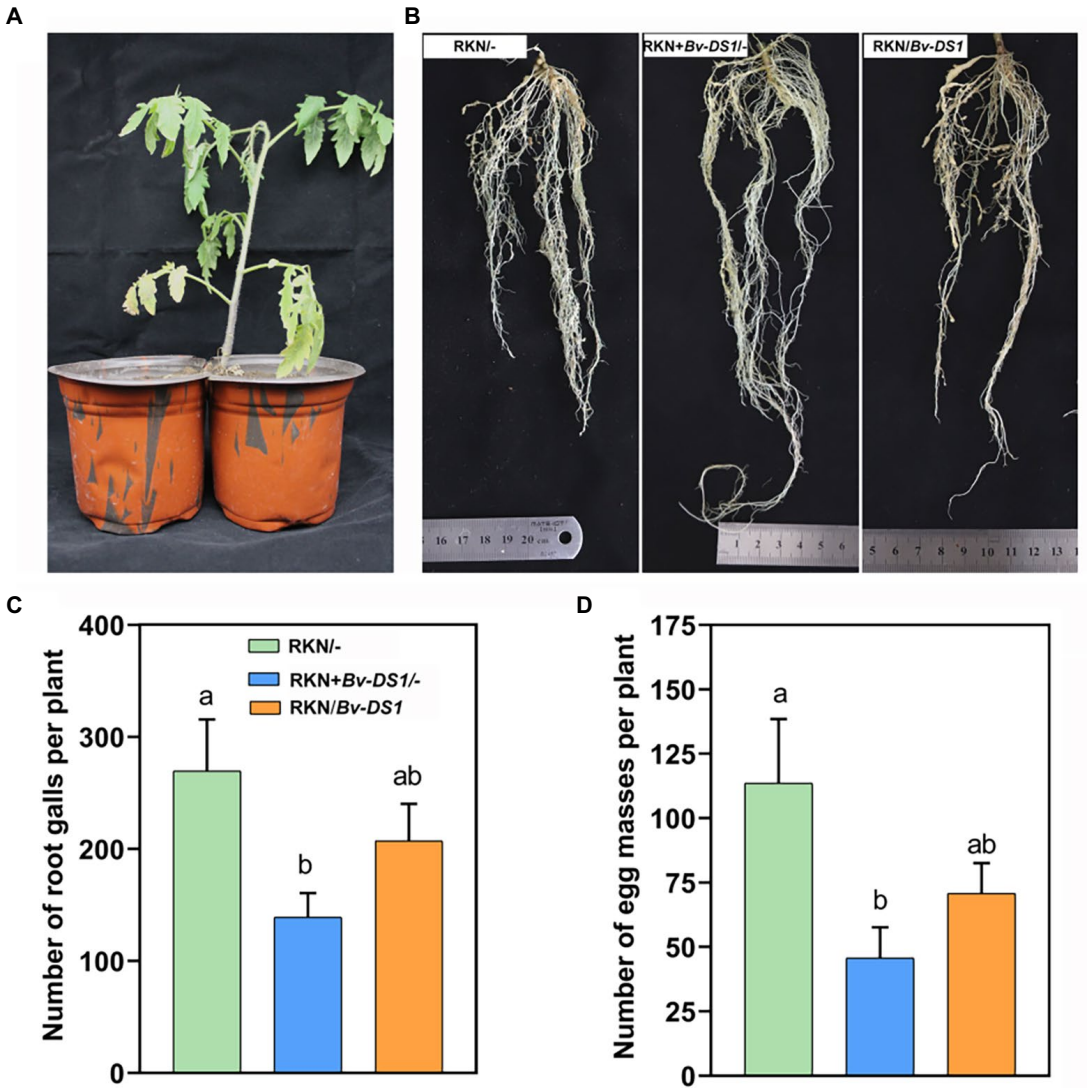
*Bacillus velezensis* YS-AT-DS1 (*Bv-DS1*) induced the local resistance of tomato plants to *M. incognita* in the split-root assay. **(A)** The split-root system of tomato growth in two adjacent pots. **(B)** Less root galls were observed in tomato plants with half of roots inoculated with *Bv-DS1* and RKN together (RKN + *Bv-DS1*/−) compared to other treatments (RKN/−; RKN/*Bv-DS1*). **(C)** The number of galls was counted at 35 dai in the split-root system. Data are shown as mean ± SE of six plants for each treatment. Different letters indicate significant differences between treatments (*p* < 0.05) according to Tukey’s multiple comparisons test following one-way ANOVA. **(D)** Number of eggs per egg mass. Data are presented as mean ± SE of six plants for each treatment. Different letters indicate significant differences between treatments (*p* < 0.05) according to Tukey’s multiple comparisons test following one-way ANOVA.

To assess whether the *Bv-DS1*-mediated plant resistance to RKN occurred in the systemic root tissue of tomato, a split-root system of tomato (Martínez-Medina et al., 2017) was used (Figure 5A). Compared to the control roots (only inoculated with RKN), pre-inoculation with *Bv-DS1* caused a reduction in the galling of the local root system (*Bv* + RKN/−) (Figure 5B). The number of galls and egg masses in the local root system of the *Bv-DS1*-treated plants significantly decreased by 48.42 and 64.81%, respectively (Figures 5C,D). Although there was a slight reduction in the number of galls and egg masses in the systematic root tissue after *Bv-DS1* treatments, no statistical significance was found in the *Bv-DS1-*induced systemic protective effects when compared to the control group (Figures 5C,D). These results indicated that *Bv-DS1* could not elicit systemic resistance to RKN in tomatoes.

### Effects of *Bv-DS1* on the defence-responsive gene expression in tomato roots

**Figure 6:**
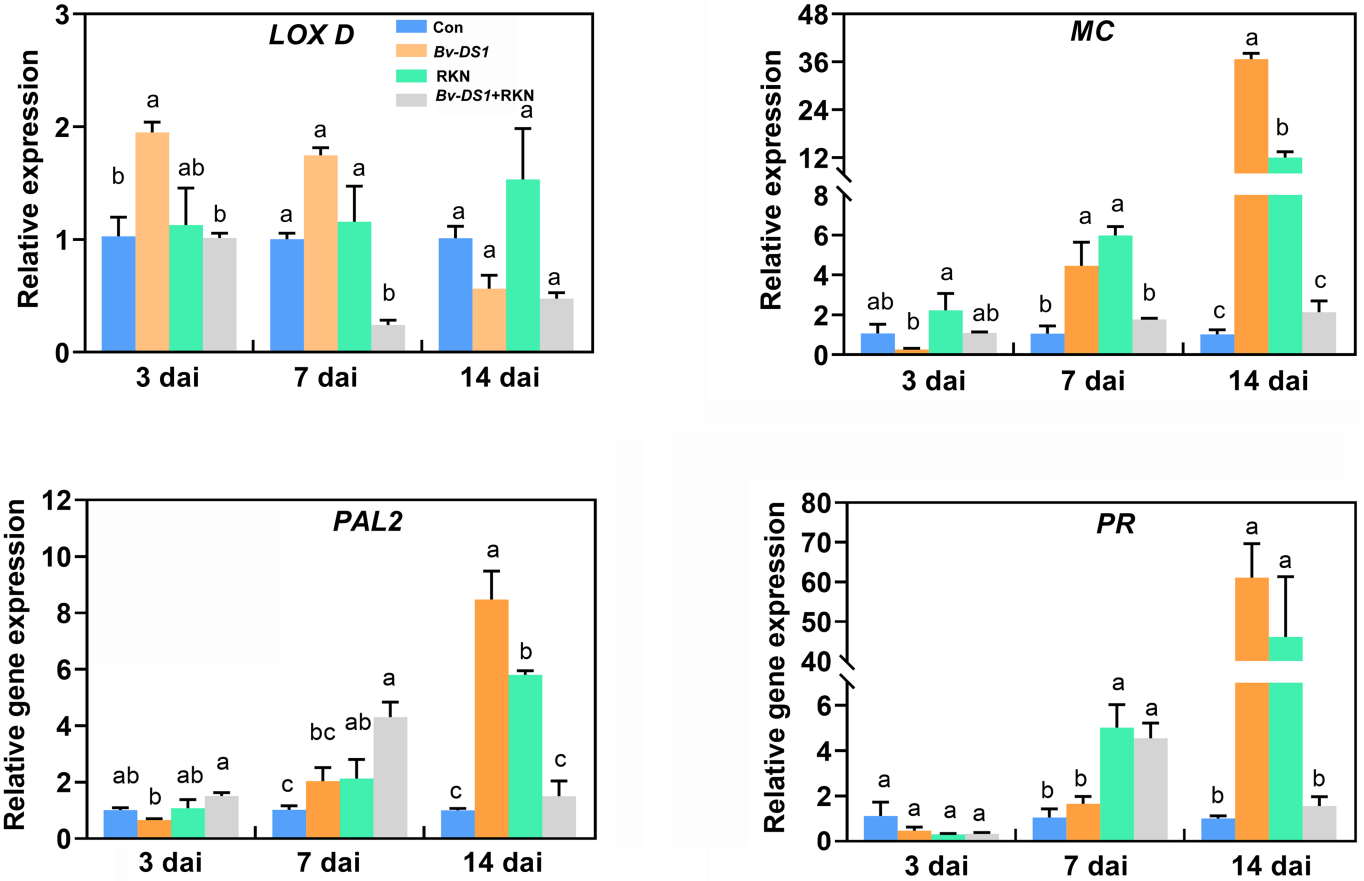
Expression levels of defence-related genes were determined in tomato roots non-inoculated or pre-inoculated with *Bacillus velezensis* YS-AT-DS1 at 3, 7, and 14 days after *M. incognita* inoculation. Relative gene expression level was normalized to the tomato reference gene *SIEF*. Error bars represent the SE of the mean of three biological replicates for three plant roots for each treatment. Different letters indicate significant differences between treatments (*p* < 0.05) according to Tukey’s multiple comparisons test following one-way ANOVA.

SA and JA are two important plant hormones and play crucial roles in plant defence response to nematode infection. To examine whether SA or JA-dependent signalling contributed to the *Bv-DS1*-mediated tomato resistance to RKN, we analysed the detailed transcript abundance of the SA and JA marker genes in *Bv-DS1*-preinoculated tomato roots under RKN stress (Figure 6). The expression of JA-related genes *LOX D* and *MC* in the roots of the RKN-untreated plants was significantly upregulated *via Bv-DS1* preinoculation. RKN infection also resulted in the upregulation of transcript levels of *MC* at 7 and 14 dai. No significant effect of *Bv-DS1* on the expression of *MC* in nematode-infected roots was found at 3, 7, and 14 dai. However, co-inoculation of RKN and *Bv-DS1* caused a transiently significant downregulation of *LOX D* expression at 7 dai (Figure 6). Expression levels of SA-responsive genes *PAL2* and *PR* in the roots of *Bv-DS1* pretreated plants were similar to that of the non-inoculated tomato roots at 3 and 7 dai. Similar to the changes in JA marker genes, *PAL2* and *PR* transcripts were significantly upregulated *via Bv-DS1* preinoculation or RKN infection alone, but this activation of RKN-induced *PAL2* and *PR* expression was not observed in *Bv-DS1* preinoculated roots at 14 dai.

The SA-deficient transgenic *NahG* tomato line, the JA-deficient mutant *spr2* and their corresponding background wild-type tomato lines ‘Castlemart’ and ‘Moneymaker’ were used to further assess the roles of SA and JA pathways in the biocontrol effects of *Bv-DS1* in tomatoes against RKN. A significant reduction in root galls was observed in both *Bv-DS1*-treated ‘Castlemart’ and JA-deficient mutant *spr2* when compared to the *Bv-DS1* non-inoculation roots. Similarly, *Bv-DS1* preinoculation also resulted in a reduction in the number of root galls in both the *NahG* tomato line and the wild-type ‘Moneymaker’ at 21 dai (Supplementary Figure 3). These findings indicated that *Bv-DS1*-induced tomato resistance against RKN was not dependent on the SA or JA pathways.

### *Bv-DS1* reverses the suppression of *TIP* genes in tomato By RKN infection

**Figure 7:**
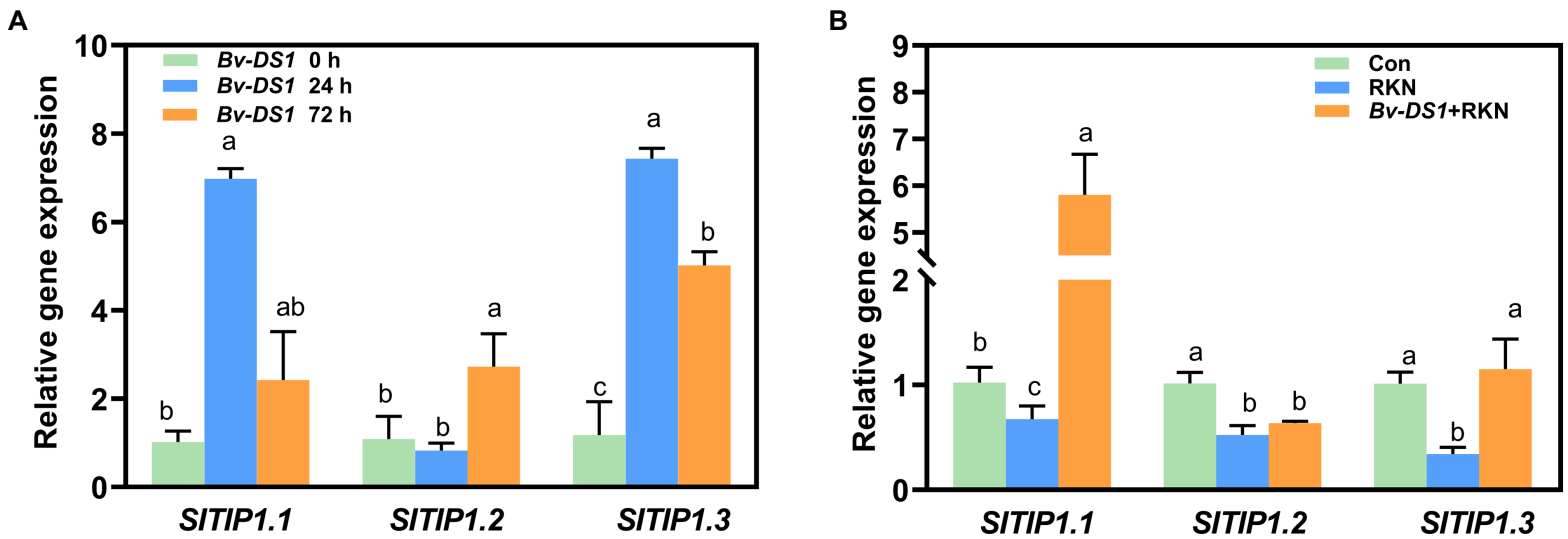
*Bacillus velezensis* YS-AT-DS1 (*Bv-DS1*) activated the expression levels of *SITIPs* in tomato roots. **(A)** Expression levels of *SITIPs* in tomato roots after *Bv*-*DS1* pre-inoculation for 0, 24, and 72 h. **(B)** *Bv*-*DS1* treatments reversed the suppression of *SITIPs* in tomato roots induced by *M. incognita* at 3 dai. Relative gene expression level was normalised to the tomato reference gene *SIEF*. Each value is presented as mean ± SE of three biological replicates for three plant roots for each treatment. Different letters indicate significant differences between treatments (*p* < 0.05) according to Tukey’s multiple comparisons test following one-way ANOVA.

Tonoplast intrinsic proteins (TIPs), localised in vacuoles, play a key role in plant defences against PPNs through the regulation of water and ion transport (Baranowski et al., 2019). Therefore, three *TIP* genes (*TIP1.1*, *TIP1.2*, *TIP1.3*), which displayed significant downregulation in the RKN-infected susceptible tomato roots (Shukla et al., 2018), were selected to study the effects of *Bv-DS*1 on their expression in the RKN-inoculated tomato roots. The expression of *TIP1.1* and *TIP1.2* was significantly upregulated by *Bv-DS1* at 24 and 72 h, respectively. *TIP1.3* transcript levels reached the peak at 24 h and then declined at 72 h but were still higher than that of untreated control roots (Figure 7A). The expression levels of three *TIP* genes were significantly downregulated at 3 dai, this suppression of RKN-induced *TIP1.1* and *TIP1.3* expression was alleviated in tomato roots through *Bv-DS1* preinoculation (Figure 7B).

## Discussion

*Bacillus velezensis* is an important member of plant growth-promoting rhizobacteria (PGPR) and are extensively studied for their potential to promote plant growth and to control soil-borne diseases (Jiang et al., 2018; Wang et al., 2020; Ding et al., 2021; Han et al., 2022). However, information about their effectivity against PPNs, including RKNs, remains limited (Xiang et al., 2017; Tian et al., 2022). This study provides evidence for the ability of a novel *B. velezensis* strain *Bv-DS1,* isolated from a tidal soil sample, to enhance tomato growth and reduce *M. incognita* infection of tomato roots. In addition, this strain also exhibited inhibitory activity against three fungal pathogens *in vitro*. Hence, the results of this study may provide valuable information to optimize the use of *Bv-DS1* as a PGPR resource for controlling a broad range of soil-borne diseases and increasing crop yield.

Numerous studies employing comparative genomic analysis have revealed that the genomes of *B. velezensis* harboured multiple gene clusters related to secondary metabolites, which are involved in plant growth promotion, biofilm formation, and antimicrobial activity (Grady et al., 2019; Xu et al., 2020; Liu et al., 2021; Zaid et al., 2022). Some reports have shown the capacity of *B. velezensis* species to form sessile communities (biofilms) (Ding et al., 2021), promoting plant growth (Xiang et al., 2017; Fan et al., 2018), and biocontrol efficacy (Wang et al., 2020; Han et al., 2022) under different experimental conditions, from *in vitro* studies to field experiments on different crops. In the present study, we found that tomato inoculation with *Bv-DS1* increased the plant height and biomass compared to untreated controls, and this PGPR trait may be related to the ability of *Bv-DS1* to produce IAA activity. The *in vitro* assay in this study suggested that *Bv-DS1* had a similar antifungal activity against soybean pathogenic fungi *R. solani* and *F. graminearum* and the genome contains several gene clusters that were predicted to be responsible for the biosynthesis of antimicrobial (surfactin, bacilysin, macrolactin, fengycin, and bacillibactin) (Supplementary Table S3). It has been shown that antibiotic substances secreted by *B. velezensis*, including surfactin, bacillomycin D, fengycin, and bacillibactin, have significant antagonistic activity against plant pathogens (Yamamoto et al., 2015; Gao et al., 2017; Chen et al., 2020). Since most of these gene clusters associated with antimicrobial activity are conserved in all *B. velezensis* strains, their antagonistic activities against pathogenic fungi and bacteria have been verified by many studies. Our research objective was focused on the biocontrol efficacy of the RKN *M. incognita*.

The results of the pot experiments demonstrated that application of *Bv-DS1* significantly reduced *M. incognita* invasion and nematode reproduction,suggesting the biocontrol potential of *Bv-DS1* in controlling PPNs. It is well known that *Bacillus* spp. can act as nematode antagonists through inhibiting J2 hatching from eggs, motility, and viability (Ghahremani et al., 2020; Chen et al., 2021; Yin et al., 2021a; Ye et al., 2022). Similar antagonism was observed in some *B. velezensis* stains, which displayed biocontrol activities against PPNs. Xiang et al. (2017) found that *B. velezensis* Bve12 can directly kill *H. glycines* J2s *in vitro*, and consistently reduced *H. glycines* population density in greenhouse and field conditions. *B. velezensis* GJ-7 can significantly suppress the hatching of *M. hapla* eggs, and the mortality rate of J2s in 100% fermentation broth of *B. velezensis* GJ-7 was 87% after 24 h treatment (Wu et al., 2022). Recently, Tian et al. (2022) isolated a PGPR strain *B. velezensis* Bv-25 from cucumber rhizosphere, which is able to disrupt the chemosensory function of *M. incognita* J2s by suppressing the expression of *ord-1* and *flp-18*. This research also pointed out that Bv-25 can inhibit egg hatching and cause J2s mortality (Tian et al., 2022). *Bv-DS1* fermentation filtrate also showed significant J2-killing activity, with the mortality rate of *M. incognita* J2s at 71.62% within 24 h treatment. We noted that almost all J2s were dead after 48 h treatment with *Bv-DS1* filtrate. This effect may contribute to the suppression of nematode infection at early stages, as well as the reduction in the number of galls and egg masses per root system in the *Bv-DS1-*inoculated pots. These studies suggested that *B. velezensis* culture filtrate may contain similar nematicidal metabolites, which are toxic to PPNs. In recent years, a large number of volatile organic compounds (VOCs) with strong nematicidal activity were identified from *Bacillus* strains (Du et al., 2017; Chen et al., 2021; Yin et al., 2021a; Ye et al., 2022). Future studies are needed to identify the VOCs with nematicidal activity from *Bv-DS1*, in order to elucidate the specific mechanism of the *Bv-DS1* strain biocontrol against *M. incognita*.

Induced systemic resistance (ISR) of the host plant is an important strategy of biocontrol microorganisms against plant pathogens. Previous studies revealed that *Bacillus* strains, including *B.velezensis,* were able to trigger ISR in nematode-infected plants that effectively reduced the disease progression (Ayaz et al., 2021; Yin et al., 2021b; Tian et al., 2022). In the current study, the split-root experiments demonstrated that pre-treatment with *Bv-DS1* in half of the tomato roots failed to significantly reduce the number of galls and egg masses on the other half of the roots inoculated with RKN. This indicates that *Bv-DS1* does not induce ISR, only local plant defences against RKN in split-root system of tomato. Our data are not in agreement with previous observations revealing the ability of *B.velezensis* Bv-25 to suppress RKN infection through the ISR mechanism in split-root system of cucumbers (Tian et al., 2022). Interestingly, the findings of Ghahremani et al. (2020) indicated that *B. firmus* can induce ISR against *M. incognita* in tomatoes but not in cucumbers in a split-root system. These observations imply that ISR in host plants by *Bacillus* spp. is dependent on the bacterial strain or plant species. It is therefore possible that the ability of *Bv-DS1* to activate the ISR would be observed in other crops when exposed to infection by other PPNs. In plant-RKN interactions, the functions of phytohormones JA and SA have been well documented for their contribution to host plant defence (Molinari et al., 2014; Kammerhofer et al., 2015). Some *Bacillus* species were able to induce plant resistance against RKN by activating JA-and/or SA signalling. For example, the expression of *PR1* and *PR3* associated with SA signalling in nematode-infected cucumber roots was induced by Bv-25 (Tian et al., 2022). Wu et al. (2022) reported that the inoculation with *B. velezensis* GJ-7 strains induced the expression of *PnPR1*, a SA marker gene, in *Panax notoginseng* roots, suggesting that SA pathway may contribute to the GJ-7-mediated *P. notoginseng* resistance against RKN. Bc-cm103 (*B. cereus* strain) promoted the expression of *LOX1* genes related to JA in cucumbers following RKN inoculation for just 6 h (Yin et al., 2021a). The upregulation of JA related genes by *B. firmus* was observed at 7 days and 40 days after RKN inoculation in tomatoes but no effect was found in cucumbers (Ghahremani et al., 2020). Nematicidal volatiles (MIV and 2-UD) from *B. atrophaeus* have been reported to upregulate the expression of JA-and SA-related genes (*PR1*, *PR5*, and *LOX1*) in tomato roots (Ayaz et al., 2021). Our results from qRT-PCR showed that individual *Bv-DS1* can trigger the upregulation of *LOX D* and *MC* expression related to JA genes at 3, 7, 14 days after preinoculation, and the SA marker genes *PAL2* and *PR* were induced from 7 to 14 days. However, the activation of SA and JA marker genes by *Bv-DS1* was not observed in tomato roots during nematode infection, suggesting that *Bv-DS1*-induced resistance against RKN is independent of the JA and SA signalling pathways. Previously, Martínez-Medina et al. (2017) used JA-and SA-impaired tomato plants to confirm the role and timing of SA-and JA pathways in *Trichoderma*-induced resistance to RKN. These transgenic and mutant tomato plants were used in this study. There was no difference in the development of RKN on SA-or JA mutants with or without *Bv-DS1* inoculation. This finding was in line with our observation of the changes in the expression of the SA and JA marker genes induced by Bv-DS1 and RKN, suggesting that JA-and SA-dependent defences were not required for *Bv-DS1-*mediated protection against RKN in tomatoes.

The tonoplast intrinsic proteins (TIPs) have been described as the most abundant aquaporin proteins localized in the plant tonoplast (Maurel et al., 2009) and play an important role in plant growth and development by regulating the transport of small substrates, such as water, glycerol, ammonia, H_2_O_2_, and urea (Höfte et al., 1992; Gerbeau et al., 1999; Soto et al., 2008; Lindahl et al., 2018). TIPs have also been found to regulate plant responses to PPN infection (Szakasits et al., 2009; Xue et al., 2013; Baranowski et al., 2019). In *Arabidopsis*, the feeding-site (syncytia) formation by cyst nematode is accompanied by reduced expression of several *TIP* genes (Szakasits et al., 2009; Baranowski et al., 2019). Among them, the characteristic downregulation of *TIP1;1* gene was further validated by observing the reduction in accumulation of γ-TIP1; 1-YFP fusion protein in nematode feeding sites (Baranowski et al., 2019), whereas *Arabidopsis* mutants *TIP1;1* exhibited increased susceptibility to *Heterodera schachtii* (Baranowski et al., 2019), suggesting that *TIP1;1* negatively regulated the parasitism of cyst nematodes. In tomatoes, transcriptome data revealed the downregulation of multiple plant aquaporins including *TIPs* upon RKN infection (Ji et al., 2013; Shukla et al., 2018). Similarly, we found that inoculation with *M. incognita* resulted in significant downregulation of *TIP1.1*, *TIP1.2*, and *TIP1.3* genes in tomato. These results indicate there is a tight relationship between *TIPs* expression and PPN parasitism in plant. Intriguingly, tomato seedlings inoculated with *Bv-DS1* had significantly upregulated expression of three *TIPs* (*TIP1.1, TIP1.2*, and *TIP1.3*). More significantly, the suppression of *TIP1.1* and *TIP1.3* expression by RKNs could be reversed using *Bv-DS1* pre-treatment, suggesting that TIPs participate in *Bv-DS1-*mediated resistance of tomatoes against RKNs. Although a *B. megaterium* strain was previously reported to regulate aquaporin proteins (ZmPIPs) in maize under salt stress (Marulanda et al., 2010), this was the first report that revealed the putative function of TIPs, the subfamily members of aquaporin, in plant resistance against RKN by *Bacillus* strains. In addition, Xue et al. (2013) reported that tomato TIP2;3 protein was hijacked by the Mi8D05 effector of *M. incognita*, which eventually might promote the giant cell development *via* control of water and solute transport. It has been proposed that the maintenance of turgor pressure in feeding cells of PPNs is very important for the nutrient sink function of nematodes (Böckenhoff and Grundler, 1994; Hofmann and Grundler, 2007). Thus, we hypothesized that the modification of tomato TIP aquaporin expression by *Bv-DS1* may interfere with the hydraulic and turgor pressure of giant cells by regulating the flux of water and solute metabolites, resulting in a suppression of RKN development. Based on our data, it is worth clarifying the role of TIPs in *Bv-DS1-*induced resistance against RKN by using a genetic method to construct TIP mutants in tomatoes.

## Conclusion

In summary, the *B*.v*elezensis* strain YS-AT-DS1 exhibited IAA production, antifungal, and nematicidal activities against *M. incognita* in tomatoes. The mechanisms details of its resistance to *M. incognita* were related with neither the ISR nor the JA-and SA-dependent pathways, but might be closely related with the regulation of water and solute transport *via* activating the expression of *TIP1.1* and *TIP1.3*, under the described conditions. Further studies are required to understand the function of aquaporin protein TIPs involved in *Bv-DS1-*mediated resistance against *M. incognita*. In addition, genome analysis illustrated that it encodes several potential genes implicated in biocontrol effects. Thus, this study provided a theoretical reference for *B. velezensis* strain commercialization as a potential candidate for eco-friendly biofertilizer. Its biocontrol effects on field crops and its potential plant growth promoting activities, and the mechanisms by which they occur, merit further investigation.

## Data availability statement

The datasets presented in this study can be found in online repositories. The names of the repository/repositories and accession number(s) can be found at: https://www.ncbi.nlm.nih.gov/, CP102866.

## Author contributions

YH and ZY conceived and designed the experiments, analyzed the data, and wrote the manuscript. YH, ZY, JY, YW, YL, SW, and FP performed the experiments. All authors have read and approved the final manuscript.

## Funding

This work was supported by the National Key R & D Program of China (2021YFD1500803), Youth Innovation Promotion Association of CAS (Nos. 2019233 and 2020236).

## Conflict of interest

The authors declare that the research was conducted in the absence of any commercial or financial relationships that could be construed as a potential conflict of interest.

## Publisher’s note

All claims expressed in this article are solely those of the authors and do not necessarily represent those of their affiliated organizations, or those of the publisher, the editors and the reviewers. Any product that may be evaluated in this article, or claim that may be made by its manufacturer, is not guaranteed or endorsed by the publisher.
