# Supplementary material for: Biocontrol efficacy of Bacillus velezensis strain YS-AT-DS1 against the root-knot nematode Meloidogyne incognita in tomato plants
Source: Front Microbiol. 2022 Nov 22;13:1035748. doi: 10.3389/fmicb.2022.1035748 (PMC9722970; doi:10.3389/fmicb.2022.1035748)
Supplement: Supplementary file 1 [file Data_Sheet_1.docx]

Supplementary Material

## Supplementary Figures

100

93

49

93

100

83

86

63

95

81

99

96

74

66

98

0.0050

*Bacillus halotolerans* ATCC 25096^T^(LPVF01000003)

*Bacillus mojavensis* RO-H-1^T^(JH600280)

*Bacillus inaquosorum* KCTC 13429^T^(AMXN01000021)

***Bacillus velezensis* YS-AT-DS1**^T^**(CP102866)**

*Bacillus velezensis* CR-502^T^(AY603658)

*Bacillus atrophaeus* JCM 9070^T^(AB021181)

*Bacillus glycinifermentans* GO-13^T^(LECW01000063)

*Bacillus paralicheniformis* KJ-16^T^(KY694465)

*Bacillus sonorensis* NBRC 101234^T^(AYTN01000016)

*Bacillus swezeyi* NRRL B-41294^T^(MRBK01000096)

*Bacillus xiamenensis* HYC-10^T^(AMSH01000114)

*Bacillus safensis* sub sp*. safensis* FO-36b^T^(ASJD01000027)

*Bacillus australimaris* NH7I_1^T^(JX680098)

*Bacillus pumilus* ATCC 7061^T^(ABRX01000007)

*Bacillus zhangzhouensis* DW5-4^T^(JOTP01000061)

*Bacillus haikouensis* C-89^T^(KJ868191)

*Bacillus salacetis* SKP7-4^T^(LC367333)

*Bacillus gobiensis* FJAT-4402^T^(CP012600)

**Supplementary Figure 1.** A phylogenetic tree based on 16S rRNA sequences obtained using the neighbour-joining method with 1,000 replicates.


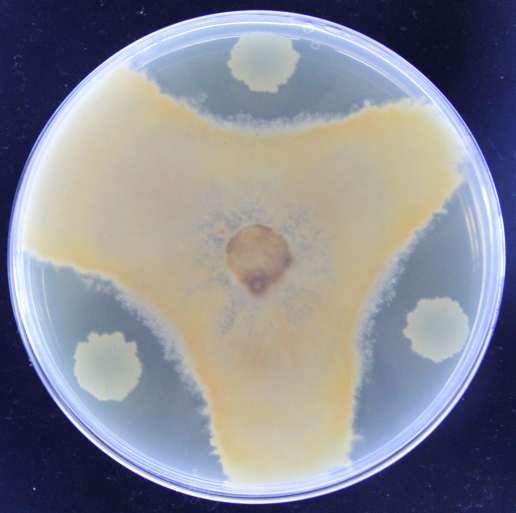

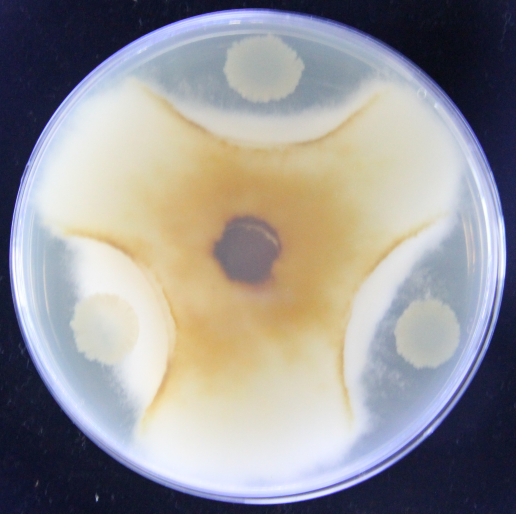

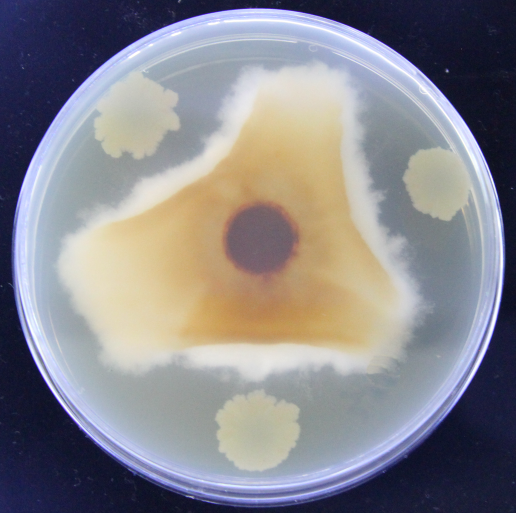


***R. solani***

***F. avenaceum***

***F. graminearum***

**Supplementary Figure 2.** Antifungal activity of *Bacillus velezensis* strain YS-AT-DS1 (Bv-DS1) against *Rhizoctonia solani*, *Fusarium avenaceum*, and *Fusarium graminearum* by plate confrontation method. Pathogens were inoculated in the centre of the plate and Bv-DS1 was inoculated at the edge of the plate (1 OD, 50 uL).


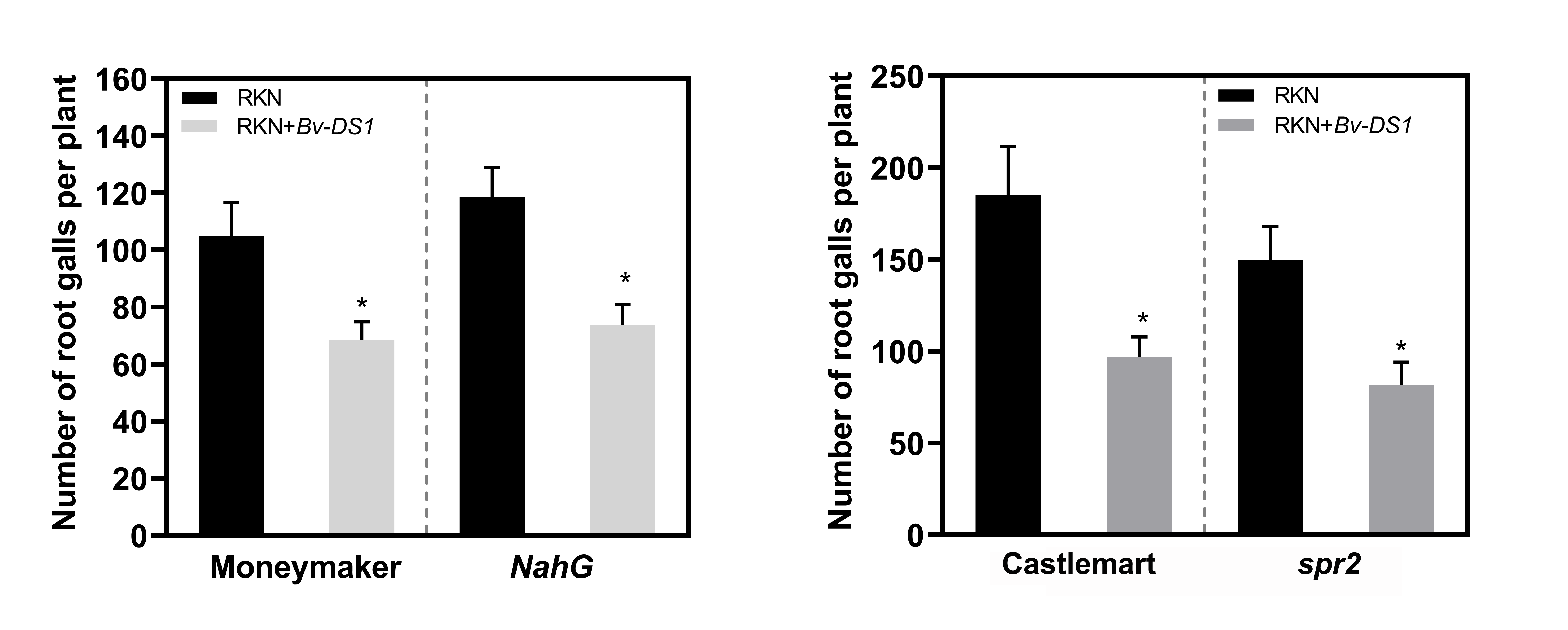


**A**

**B**

**Supplementary Figure 3**. (**A)** The effects of *Bacillus velezensis* YS-AT-DS1 (*Bv-DS1*) on root-knot formation in the wild-type plant (Moneymaker) and SA-deficient transgenic line (*NahG*). (**B**) The impacts of *Bacillus velezensis* YS-AT-DS1 on root-knot formation in the wild-type tomato plant (Castlemart) and JA-related mutant (*spr2*). Number of galls was counted per pre-inoculated or non-inoculated tomato plant with *Bv-DS1* at 21 days after nematode inoculation. Data are shown as means ± SE of six plants for each treatment. Asterisk indicates significant differences between treatments (*p* < 0.05) according to Student’s *t* test.

## Supplementary Tables

**Supplementary Table 1.** Primer sequences used for qRT-PCR analysis.

| Gene Name | ID | Primer sequences (5'- 3') | References |
| --- | --- | --- | --- |
| Pathogenesis-related protein 1a (*PR1a*) | M69247 | Forward: ATGTGTGTGTTGGGGTTGGT;  Reverse: ACTTTGGCACATCCAAGACG | López-Ráez et al., 2010 |
| Phenylalanine ammonia lyase (*PAL2*) | TC165415 | Forward: TGAAGGAATGGAATGGTGCT;  Reverse: TGAAAGAAGCCACAAAAGTTCA | Gayoso et al. 2010 |
| *M. incognita* Actin  (*Min-Actin*) | MINC06773a | Forward: GATGGCTACAGCTGCTTCGT;  Reverse: GGACAGTGTTGGCGTAAAGG | Teillet *et al*., 2013 |
| Lipoxygenase D (*LoxD*) | U37840 | Forward: GACTGGTCCAAGTTCACGATCC;  Reverse: ATGTGCTGCCAATATAAATGGTTCC | Uppalapati et al., 2005 |
| Multicystatin (*MC*) | AF083253 | Forward: GAGAATTTCAAGGAAGTTCAA;  Reverse: GGCTTTATTTCACACAGAGATA | Uppalapati et al., 2005 |
| Elongation factor 1α (*EF1α*) 4 | X14449 | Forward: GATTGGTGGTATTGGAACTGTC;  Reverse: AGCTTCGTGGTGCATCTC | Rotenberg et al., 2006 |
| Tonoplast intrinsic protein 1.1 (*TIP1.1*) | Solyc10g083880 | Forward: GAAGGAGGGAGGAAGCCACT;  Reverse: CCAACCGAAACCGCTACGA |  |
| Tonoplast intrinsic protein 1.2 (*TIP1.2*) | Solyc06g074820 | Forward: GTCAGGGTTCTGGTATGGC;  Reverse: AAGGTAACAGCAGGGTTGACG |  |
| Tonoplast intrinsic protein 1.3 (*TIP1.3*) | Solyc06g075650 | Forward: GCTGGTTCAGGATCTGGCAT;  Reverse: CGGCAGGATTTACGTGACCT |  |

**Supplementary Table 2. Genome characteristics of *Bacillus velezensis* YS-AT-DS1**

| **Genome characteristics** | **Value** |
| --- | --- |
| Genome Size (Mbp) | 4.73 |
| Topology | circular |
| Total length (bp) | 4,007,438 |
| GC content (%) | 46.43 |
| Protein coding genes (CDS) | 3,977 |
| tRNA | 86 |
| rRNA (5S, 16S, 23S) | 9, 9, 9 |
| Prophage | 8 |
| Genes allocated to COG | 2,891 |

**Supplementary Table 3. Gene cluster type, location, compound, and size of *Bacillus velezensis* YS-AT-DS1**

| **Cluster no** | **Type** | **From** | **To** | **Most similar known cluster** | **Size (nt)** | **Similarity** |
| --- | --- | --- | --- | --- | --- | --- |
| 1 | NRPS | 317,578 | 382,671 | Surfactin | 65,093 | 78% |
| 2 | T1PKS, TransAT-PKS, T3PKS | 661,949 | 772,753 | Aurantinin B/Aurantinin C/Aurantinin D | 110,804 | 17% |
| 3 | PKS-like | 992,884 | 1,034,128 | Butirosin A/Butirosin B Saccharide | 41,244 | 7% |
| 4 | Terpene | 1,119,730 | 1,137,123 | - | 17,393 | - |
| 5 | Trans ATPKS | 1,416,684 | 1,504,505 | Macrolactin H Polyketide | 87,821 | 100% |
| 6 | Trans ATPKS, T3PKS, NRPS | 1,724,012 | 1,824,556 | Bacillaene Polyketide + NRP | 100,544 | 100% |
| 7 | NRPS, Trans ATPKS, Betalactone | 1,896,441 | 2,030,433 | Fengycin | 133,992 | 100% |
| 8 | Terpene | 2,059,109 | 2,080,992 | - | 21,883 | - |
| 9 | T3PKS | 2,170,141 | 2,211,292 | - | 41,151 | - |
| 10 | Trans ATPKS | 2,382,174 | 2,475,966 | Difficidin | 93,792 | 100% |
| 11 | NRPS, RiPP-like | 3,092,497 | 3,144,287 | Bacillibactin | 51790 | 100% |
| 12 | Other | 3,666,162 | 3,707,580 | Bacilysin | 41,418 | 100% |
